# Evaluation of blood cellular and biochemical parameters in rats under a chronic hypoxic environment at high altitude

**DOI:** 10.1080/07853890.2023.2184859

**Published:** 2023-03-10

**Authors:** Chunlong Yan, Dengfeng Tian, Chenhong Zhang, Qiang Zhang, Yanqiu Sun

**Affiliations:** a Suzhou Medical College of Soochow University, suzhou, China; b Department of Radiology, Jining No.1 People’s Hospital, Jining, China; c Department of Radiology, Qinghai Provincial People’s Hospital, Xining, China; d Department of Neurosurgery, Qinghai Provincial People's Hospital, Xining, China

**Keywords:** High altitude, low hypoxia, red blood cells, white blood cells, platelets, blood biochemistry

## Abstract

**Background:**

The purpose of this study was to explore the changes in blood cellular and biochemical parameters of rats in a natural environment of low pressure and low oxygen on the plateau.

**Methods:**

Male Sprague-Dawley rats in two groups were raised in different environments from 4 weeks of age for a period of 24 weeks. They were raised to 28 weeks of age and then transported to the plateau medical laboratory of Qinghai University. Blood cellular and biochemical parameters were measured and the data of the two groups were statistically analyzed.

**Results:**

1. RBC in the HA group was higher than that in the Control group, but there was no significant difference between the two groups (*p* > 0.05), Compared with the Control group, HGB, MCV, MCH, MCHC and RDW in the HA group were significantly higher (*p* < 0.05). 2. Compared with the Control group, WBC, LYMP, EO, LYMP% and EO% in the HA group decreased significantly (*p* < 0.05), and ANC% increased significantly (*p* < 0.05). 3. In the platelet index, compared with the Control group, PLT in the HA group was significantly reduced (*p* < 0.05), PDW, MRV, P-LCR were significantly increased (*p* < 0.05). 4. In blood biochemical indicators, compared with the Control group, AST, TBIL, IBIL, LDH in the HA group decreased significantly (*p* < 0.05), CK in the HA group increased significantly (*p* < 0.05).

**Conclusions:**

1. The indexes related to red blood cells, white blood cells, platelets and some biochemical indexes in the blood of rats at high altitude have changed. 2. Under the high altitude environment, the oxygen carrying capacity of SD rats is improved, the resistance to disease may be reduced, the coagulation and hemostasis functions may be affected, and there is a risk of bleeding. The liver function, renal function, heart function and skeletal muscle energy metabolism may be affected. 3. This study can provide an experimental basis for the research on the pathogenesis of high-altitude diseases from the perspective of blood.

**KEY MESSAGES**
- In this study, red blood cells, white blood cells, platelets and blood biochemical indicators were included in the real plateau environment to comprehensively analyze the changes of blood cellular and biochemical parameters in rats under the chronic plateau hypobaric hypoxia environment.
- From the perspective of blood, this study can provide an experimental basis for research on the pathogenesis of high-altitude diseases.
- Explore the data support of oxygen-carrying capacity, disease resistance and energy metabolism of the body in the natural environment at high altitude.

## Introduction

Plateau areas are characterized by low pressure, low humidity, large temperature differences between day and night, and strong ultraviolet radiation [1–2], which affect multiple systems of the body. However, the pathogenesis of high-altitude disease has not been fully clarified [3–4]. In addition, it has been reported in previous studies that in the high-altitude, low-pressure environment, the oxygen content in the arterial blood of the body is reduced, which leads to a high level of expression of erythropoietin, thus increasing the red blood cells and haemoglobin content to improve the oxygen-carrying capacity [5–6]. Furthermore, according to a literature review, there are many reports on red blood cells and platelets as blood cellular parameters, but there are few reports on other blood cellular parameters. Moreover, experimental studies on animals at high altitudes are mostly conducted in simulated spaces such as low-pressure and hypoxic chambers [7–8], which cannot objectively and truly reflect the physiological and pathological changes of the subjects at high altitudes. Therefore, in this study, red blood cells, white blood cells, platelets and blood biochemical indicators were included in the real high-altitude hypobaric hypoxic environment to comprehensively analyze the changes in blood cellular and biochemical parameters of rats in a chronic high-altitude hypobaric hypoxic environment.

## Materials and methods

SPF Sprague–Dawley (SD) rats (males, *n* = 20, 4 weeks old) were purchased from Chengdu Dashuo Laboratory Animal Company (production license number: SCXK (Chuan) 2020-030; use license number: SYXK (Chuan) 2018-119), as approved by the Medical Ethics Committee of Qinghai Provincial People’s Hospital. The animal treatment adopted the ‘Guiding Opinions on Treating Laboratory Animals Kindly’ issued by the Ministry of Science and Technology in 2006. The study adhered to the ARRIVE guidelines. This study was approved by the Medical Ethics Committee of Qinghai Provincial People’s Hospital (2020-115).

### Numbering and grouping of experimental animals

The experimental animals were numbered and marked using the number plate method, where a metal number plate was fixed on the ears of the rats. SD rats were transported to the Chengdu area (approximately 500 m above sea level) and Yushu area (approximately 3800 m above sea level) for rearing. Rats in the Chengdu area were defined as the Control group, and rats in the Yushu area were defined as the High Altitude (HA) group. There were 10 rats in the Control group and 10 rats in the HA group. The rats in the Control group and the HA group were raised to 28 weeks of age.

### Laboratory animal feeding environment and management

The experimental animals were raised in animal laboratories in the plateau and plain areas. The indoor air of the animals was kept ventilated, the circadian rhythm of natural light was changed, the temperature of the mouse room was 18–25 °C, the humidity was 40–60%, and the mice were fed with sufficient feed every day.

### Blood cellular and biochemical parameters detection

The rats were transported to the Plateau Medical Laboratory of Qinghai University, weighed, and anesthetized intraperitoneally with 10% chloral hydrate according to the standard of 1 ∼ 2ml/100g. After the rats were completely anesthetized, they were placed on an operating table of moderate size. The tail vein puncture blood collection method was used to collect rat blood; this required inhalation anesthesia, where the anesthetic gas was set to a mixture of 2% to 3% isoflurane and oxygen. After the rat was anesthetized the blood volume (1–2 mL) was collected using the venous trocar catheter for animal-specific venous blood collection with a vacuum blood collection tube. Detect red blood cell-related indicators (RBC, HGB, HCT, MCV, MCH, MCHC, RDW), white blood cell-related indicators (WBC, ANC, LYMP, MONO, EO, BASO, ANC%, LYMPH%, MONO%, EO%, BASO%) and blood biochemical indicators (ALT, AST, ALT/AST, TBIL, DBIL, IBIL, GGT, ALP, Cr, CK, LDH, TC). Blood cellular and biochemical parameters were determined using a Coulter Automated Cell Counter (Coulter AcT, Beckman Coulter, New York, NY, USA).

### Statistical processing

SPSS 25.0 statistical software was used for the statistical processing of data. Measurement data that conformed to a normal distribution, expressed as the mean ± standard deviation ($\bar{x}$±s), were analyzed by independent samples *t* tests. Measurement data that did not conform to a normal distribution were expressed using the median and quantile (interquartile range, M (IQR)) and were analyzed by the Mann–Whitney *U* test. The test level was *a* = 0.05, and the difference was considered statistically significant when *p* ≤ 0.05.

## Results

**Figure 1.**
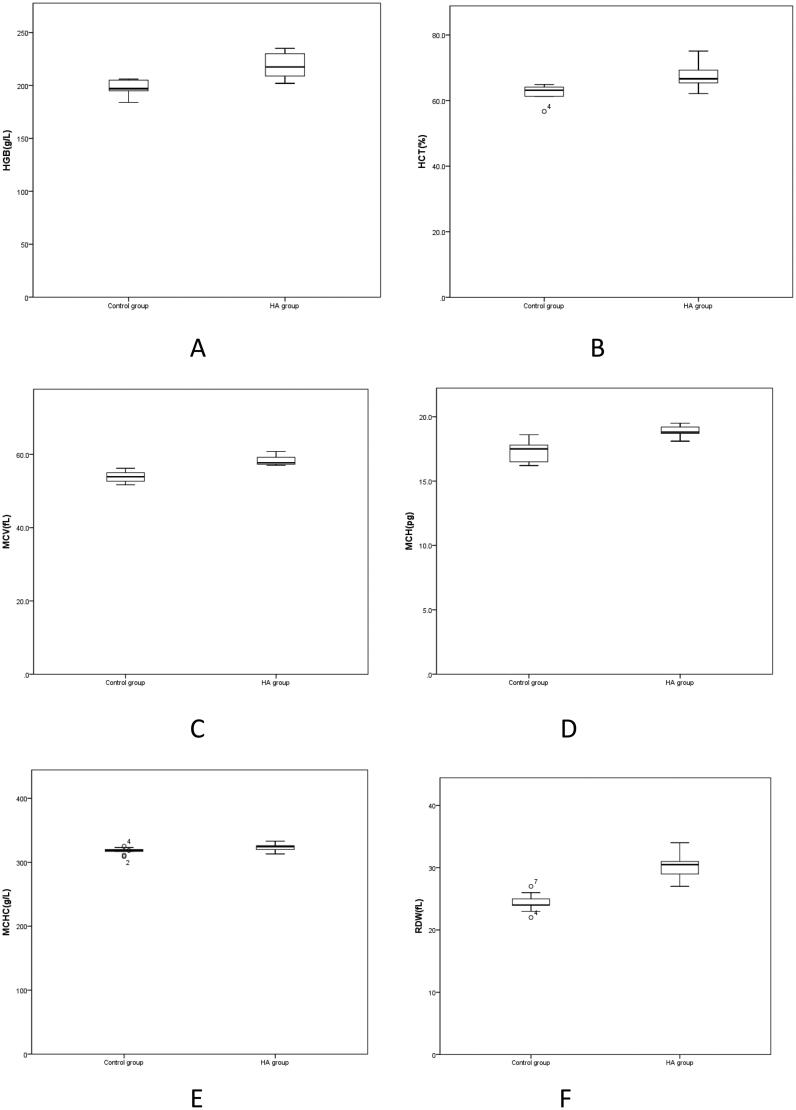
A-F Comparison of erythrocyte parameters between HA group and Control group. HGB: Hemoglobin; HCT: Hematocrit; MCV: mean corpuscular volume; MCH: mean corpuscular hemoglobin; MCHC: mean corpuscular hemoglobin concentration. RDW: red blood cell volume distribution width.

1. According to Table 1, the body weight in the Control group was higher than that in the HA group, but there was no significant difference between the two groups (*p* > 0.05). RBC in the HA group was higher than that in the Control group, but there was no significant difference between the two groups (*p* > 0.05). Compared with the Control group, HGB, MCV, MCH, MCHC, RDW in the HA group were significantly increased (*p* < 0.05) (Figure 1(A–F)).

**Table 1.** Comparison of body weight and erythrocyte parameters between the HA group and the Control group.

Index	Control group (*n* = 10)	HA group (*n* = 10)	Test statistics	*p* value
Body weight (g)	659.00 ± 53.43	656.00 ± 48.12	0.132^a^	0.896
RBC (10^12^/L)	11.59 ± 0.57	11.60 ± 0.66	−0.047^a^	0.963
HGB(g/L)	197.80 ± 7.17	218.40 ± 11.06	−4.942^a^	<0.001
HCT (%)	62.38 ± 2.38	67.63 ± 3.55	−3.884^a^	0.001
MCV (fL)	53.89 ± 1.47	58.24 ± 1.23	−7.159^a^	<0.001
MCH (pg)	17.31 ± 0.81	18.85 ± 0.44	−5.274^a^	<0.001
MCHC (g/L)	318.10 ± 4.89	322.90 ± 5.78	−2.005^a^	0.06
RDW (fL)	24.35 ± 1.43	30.14 ± 1.99	−7.476^a^	<0.001

Note: ^a^is t value.

**Figure 2.**
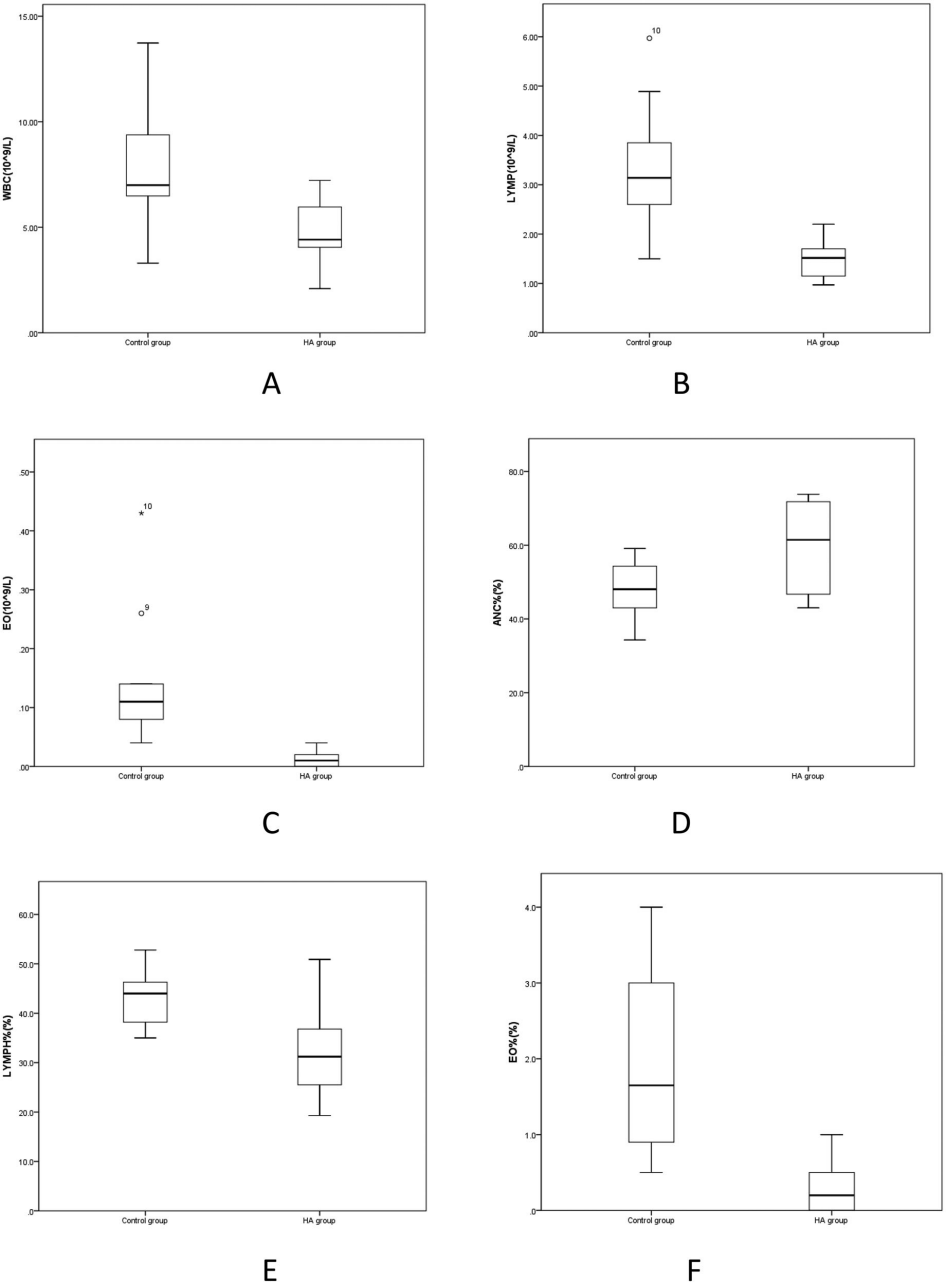
A-F Comparison of leukocyte parameters between HA group and Control group. WBC: White blood cell count; LYMP; Absolute number of lymphocytes; EO: Absolute number of eosinophils; ANC%: Absolute number of neutrophils; LYMPH%: Percentage of lymphocytes.

2. According to Table 2, compared with the Control group, WBC, LYMP, EO, LYMP% and EO% in HA group were significantly decreased (*p* < 0.05), while ANC% was significantly increased (*p* < 0.05) (Figure 2(A–F)). ANC, MONO and BASO in the HA group were lower than those in the Control group, but the difference was not statistically significant (*p* > 0.05). MONO% and BASO% in the HA group were higher than those in the Control group, and the difference was not statistically significant (*p* > 0.05).

**Table 2.** Comparison of leukocyte indexes between the HA group and the Control group.

Index	Control group (*n* = 10)	HA group (*n* = 10)	Test statistics	*p* value
WBC(10^9^/L)	7.00(5.65,10.29)	4.41(3.72,5.95)	−2.343^b^	0.019
ANC (10^9^/L)	3.81 ± 1.55	2.97 ± 1.29	1.314^a^	0.205
LYMP(10^9^/L)	3.14(2.48,4.30)	1.52(1.18,1.79)	−3.402^b^	0.001
MONO(10^9^/L)	0.36(0.06,1.17)	0.30(0.14,0.32)	−0.529^b^	0.597
EO(10^9^/L)	0.11(0.06,0.23)	0.01(0.01,0.02)	−3.763^b^	<0.001
BASO(10^9^/L)	0.01(0.01,0.02)	0.01(0.01,0.01)	−1.589^b^	0.112
ANC%(%)	47.89 ± 7.71	59.77 ± 11.77	−2.670^a^	0.016
LYMPH%(%)	43.40 ± 5.73	32.65 ± 10.20	2.905^a^	0.009
MONO%(%)	4.95(2.80,10.52)	6.00(3.25,10.97)	−0.454^b^	0.650
EO%(%)	1.65(1.04,2.71)	0.20(0.07,0.53)	−3.451^b^	0.001
BASO%(%)	0.15(0.06,0.30)	0.20(0.07,0.27)	−0.081^b^	0.936

Note: ^a^is t value.

^b^is Z value.

**Figure 3.**
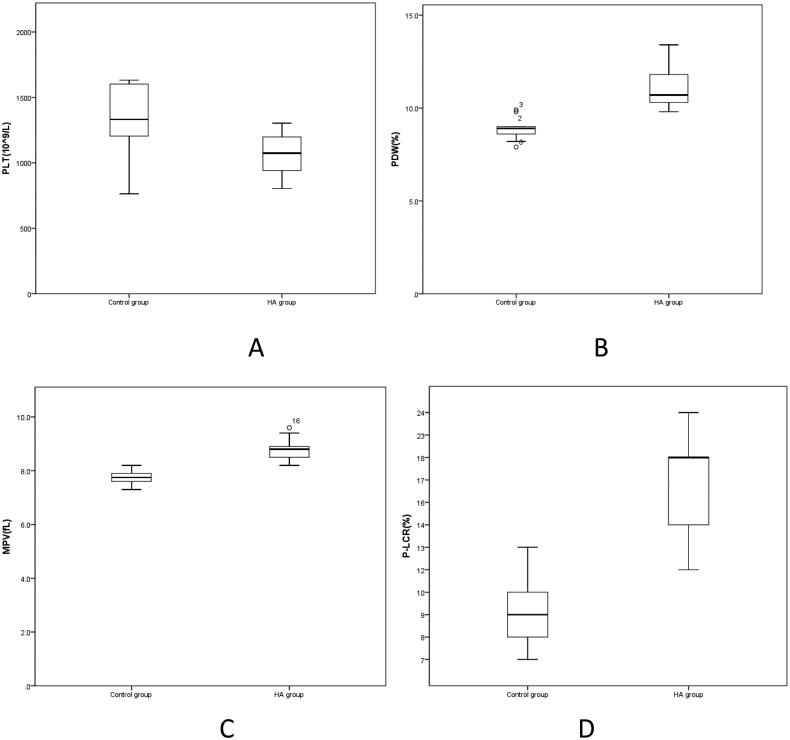
A-D Comparison of platelet parameters between HA group and Control group. PLT: Platelet count; PDW: Platelet Volume Distribution width; MPV: Mean Platelet Volume; P-LCR: Large platelet ratio.

3. According to Table 3, compared with the Control group, PLT in the HA group was significantly decreased (*p* < 0.05) (Figure 3(A)), while PDW, MRV and P-LCR were significantly increased (*p* < 0.05) (Figure 3(B–D)). PCT in the HA group was lower than that in the Control group, but the difference was not statistically significant (*p* > 0.05).

**Table 3.** Comparison of platelet indexes between HA group and Control group.

Index	Control group (*n* = 10)	HA group (*n* = 10)	Test statistics	*p* value
PLT (10^9^/L)	1338.70 ± 270.57	1069.20 ± 158.08	2.720^a^	0.014
PCT (%)	1.04 ± 0.20	1.02 ± 0.13	0.293^a^	0.773
PDW (%)	8.90 ± 0.61	11.08 ± 1.12	−5.362^a^	<0.001
MPV (fL)	7.78 ± 0.27	8.80 ± 0.44	−6.200^a^	<0.001
P-LCR (%)	9.17 ± 1.92	17.24 ± 3.92	−5.850^a^	<0.001

Note: ^a^is t value.

**Figure 4.**
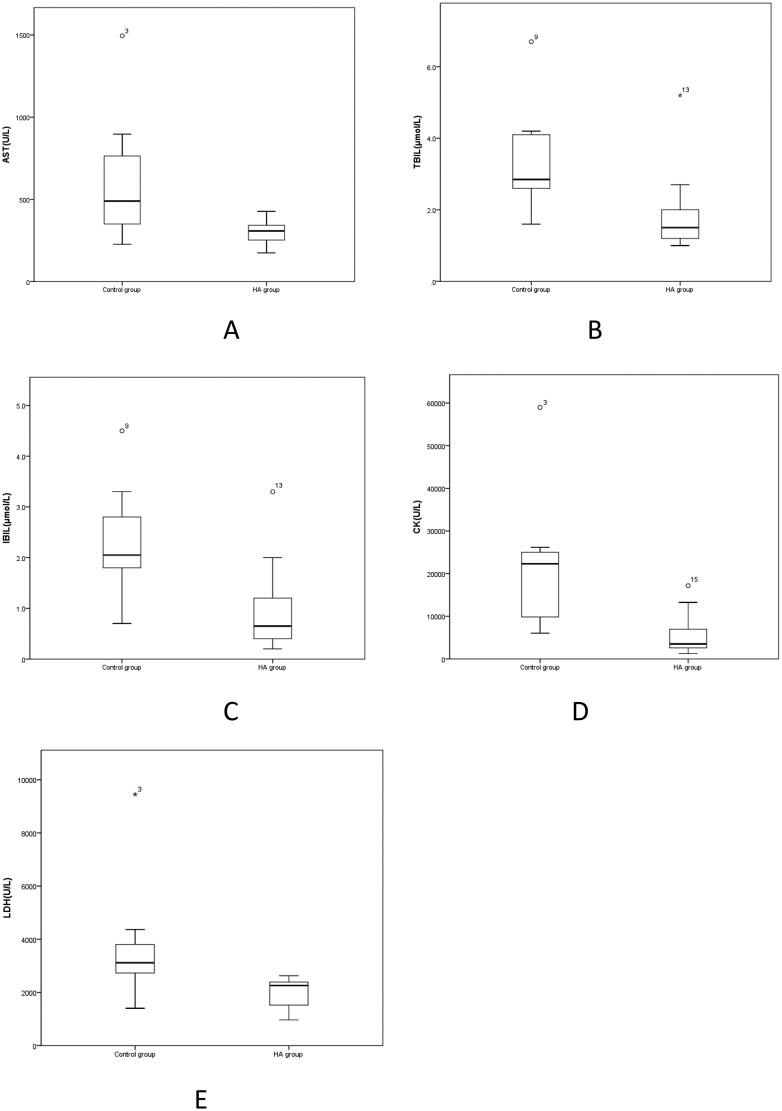
A-E Comparison of blood biochemical parameters between HA group and Control group. AST: Aspartate amino transferase; TBIL: Total bilirubin; IBIL: Indirect bilirubin; LDH: Lactate dehydrogenase.

4. According to Table 4, compared with Control group, AST, TBIL, IBIL and LDH were significantly decreased in the HA group (*p* < 0.05) (Figure 4(A–C,E)), CK was significantly increased in the HA group (*p* < 0.05) (Figure 4(D)), ALT, DBIL, Cr and TC were decreased in the HA group (*p* > 0.05), ALT/AST and ALP were increased in the HA group. There was no significant difference (*p* > 0.05).

**Table 4.** Comparison of blood biochemical indexes between the HA group and the Control group.

Index	Control group (*n* = 10)	HA group (*n* = 10)	Test statistics	*p* value
ALT (U/L)	129.00 (81.96,219.84)	76.50 (51.55,139.25)	−1.323^b^	0.186
AST (U/L)	490.00 (349.62,888.98)	308.00 (242.98,363.02)	−2.343^b^	0.019
ALT/AST	0.20 (0.16,0.34)	0.25 (0.16,0.51)	−0.760^b^	0.447
TBIL (μmol/L)	2.85 (2.31,4.32)	1.50 (1.00,2.82)	−2.577^b^	0.010
DBIL (μmol/L)	0.90 (0.70,1.36)	0.80 (0.56,1.16)	−1.104^b^	0.270
IBIL (μmol/L)	2.29 ± 1.06	1.05 ± 0.95	2.756^a^	0.013
ALP (U/L)	80.00 (58.68,122.32)	109.00 (95.88,115.72)	−1.778^b^	0.075
Cr (μmol/L)	65.40 ± 12.05	63.40 ± 15.97	0.316^a^	0.756
CK (U/L)	22309.50 (11175.84,32610.16)	3514.00 (1900.45,9528.55)	−3.250^b^	0.001
LDH (U/L)	3114.00 (1987.17,5214.23)	2258.00 (1610.12,2432.88)	−2.646^b^	0.008
TC (μmol/L)	2.42 ± 0.36	2.35 ± 0.96	0.204^a^	0.841

Note: ^a^is t value.

^b^is Z value.

## Discussion

According to the statistics of the World Health Organization (WHO), the total area of the global plateau is approximately 30 million square kilometres, and more than 140 million people live in plateau areas that are greater than 2500 metres above sea level, of which 60 million live in plateau areas of China [9]. In addition, the plateau area is characterized by low oxygen, low humidity, a large temperature difference between day and night, and strong ultraviolet radiation [1–2], among which low oxygen are the most important characteristics that affect the health of local people and restrict local development and long-term economic development. Furthermore, as a special ecological environment system, the plateau environment is highly valued by all countries for special political, social, economic, military and other reasons. However, people who migrate to or live in high-altitude areas are prone to acute or chronic high-altitude disease, multiple systems of the body are affected, and blood cellular and biochemical parameters change. As we know, there would be a period of plateau acclimation in plateau environment. In addition, it has been reported that the structure and function of the right heart of rats in chronic plateau environment for 12 weeks were studied by 7.0 T MR, RBC count, HGB and HCT were found to be significantly increased, and the structure and function of the right heart were changed [5]. However, the pathogenesis of this disease has not been fully clarified [3–4] and there are no relevant literature reports on the specific time of high altitude habitations of rats. Additionally, most of the experimental studies on animals at high altitudes are carried out in simulated spaces such as low hypoxic cabins, which cannot objectively and truly reflect the physiological and pathological changes of the subjects at high altitudes. Therefore, this study conducted research on SD rats in a real low hypoxic environment at a high altitude for a period of 24 weeks, including assessment of red blood cells, white blood cells, platelets and blood biochemical indicators, to comprehensively analyze the changes in blood indices in rats in a chronic high-altitude environment.

Red blood cells are blood cells with the largest number in the blood. In addition, they are also the main medium for vertebrates to transport oxygen through the blood. At the same time, they also have immune functions [10]. Red blood cells contain haemoglobin, which can combine with oxygen in the air. Therefore, red blood cells can transport oxygen inhaled into the alveoli to the tissues through haemoglobin, and part of the carbon dioxide generated by metabolism in the tissues is also transported to the lungs through red blood cells for the exchange of gas with oxygen outside the alveoli and the expulsion of carbon dioxide from the body [11–12]. Table 1 shows that RBCs in the HA group were higher than those in the Control group, interestingly, there was no significant difference between the two groups (*p* > 0.05). This may indicate that high-altitude acclimation has occurred in rats after 24 weeks in a high-altitude environment. Compared with the Control group, HGB, MCV, MCH, MCHC and RDW in the HA group were significantly higher (*p* < 0.05) (Figure 1(A–F)). These results show that the blood composition of rats in the HA group has changed, with increases in HGB, the percentage of red blood cells in a certain volume of whole blood, the volumes of the individual red blood cells, the average amount of haemoglobin contained in each red blood cell, the average grams of haemoglobin contained in each litre of blood cells, and the volume heterogeneity of the peripheral red blood cells. This may suggest that the decrease of oxygen in the body’s arterial blood in the altitude hypoxia environment leads to the high expression of erythropoietin and then the increase of hemoglobin to improve oxygen carrying capacity.

White blood cells are a very important kind of blood cell in the blood. In addition, White blood cells are the ‘guardians’ of the human body in the fight against the disease. When bacteria invade the human body, white blood cells can pass through the capillary wall through deformation, concentrate on the invasion site of bacteria, and surround and engulf the bacteria [13–14]. Different kinds of white blood cells participate in the body’s defence response in different ways [15–16]. Table 2 shows that compared with the Control group, WBC, LYMP, EO, LYMP% and EO% in the HA group decreased significantly (*p* < 0.05), and ANC% increased significantly (*p* < 0.05). The ANC, MONO and BASO in the HA group were lower than those in the Control group, but the difference was not statistically significant (*p* > 0.05) (Figure 2(A–F)). The MONO% and BASO% in the HA group were higher than those in the Control group, and the difference was not statistically significant (*p* > 0.05). The white blood cells in the blood components of rats at high altitudes changed, there were increases in the number of white blood cells, the absolute number of lymphocytes, the percentage of lymphocytes, the absolute number of eosinophils, and the percentage of eosinophils and a decrease in the percentage of neutrophils. The changes in leukocyte-related indicators in the HA group suggest that the body’s resistance to disease is reduced in the low hypoxic environment at high altitudes. Whether this is related to the occurrence of acute and chronic high-altitude disease remains to be further studied.

Platelets are small pieces of cytoplasm detached from the cytoplasm of mature megakaryocytes in the bone marrow [17]. In addition, the main function of platelets is to coagulate and stop bleeding and to repair damaged blood vessels [18–19]. Table 3 shows that compared with the Control group, PLT in the HA group decreased significantly (*p* < 0.05) (Figure 3(A)), PDW, MRV, and P-LCR increased significantly (*p* < 0.05) (Figure 3(B–D)), and PCT in the HA group decreased compared with the Control group, but the difference was not statistically significant (*p* > 0.05). These results show that the composition of platelets in the blood of rats at high altitude changes, with a decrease in platelet count and increases in the distribution of platelet size in the blood, the average platelet volume, and the percentage of large platelets in total blood platelets. This may indicate that coagulation and hemostasis functions may be affected in the low hypoxic environment at high altitudes, with a risk of bleeding.

In addition to blood cells, there are many different substances in the blood. Determination of the content of various ions, sugars, lipids, proteins, enzymes, hormones and various metabolites of the body in the blood is known as blood biochemical examination. Additionally, ALT and AST are indicators of liver function [20–21]. The determination of serum total bilirubin is an important test for the examination of liver and biliary function. The liver plays an important role in the metabolism of bilirubin [22]. Creatine kinase mainly exists in the cytoplasm and mitochondria. It is an important kinase directly related to energy transfer, muscle contraction and ATP regeneration in cells. The determination of creatine kinase activity can be used for the diagnosis of skeletal muscle diseases and myocardial diseases [23]. Table 4 shows that compared with the Control group, ALT, DBIL, UCr and TC in the HA group decreased, with no statistically significant difference (*p* > 0.05), ALT/AST and ALP in the HA group increased, with no statistically significant difference (*p* > 0.05), AST, TBIL, IBIL and LDH in the HA group decreased significantly (*p* < 0.05) (Figure 4(A–C,E)), and CK in the HA group increased significantly (*p* < 0.05) (Figure 4(D)). These results show that the biochemical indices in the blood of rats at high altitude were partially changed, there were decreases in aspartate aminotransferase, total bilirubin, indirect bilirubin, and lactate dehydrogenase and an increase in creatine kinase. This may indicate that the liver function, renal function, heart function and skeletal muscle energy metabolism of rats may be affected in a high-altitude hypoxic environment.

The disadvantage of this study is that the sample size included was small and will be further increased in the future. Only the changes in blood cellular and biochemical parameters of rats in the natural environments of plateaus and plains were studied. In the next step, the changes in blood cellular and biochemical parameters of rats in the natural environment at different altitudes and at different weeks of age will be further studied.

## Conclusion

In summary, the indices related to red blood cells, white blood cells, platelets and some biochemical parameters changed in the blood of rats in the high-altitude low hypoxic environment. The oxygen-carrying capacity of the body was improved, but the resistance to disease may be reduced, the coagulation and haemostasis functions may be affected, and there may be a risk of bleeding. Liver function, renal function, heart function and skeletal muscle energy metabolism may be affected. This study explored the changes in blood cellular and biochemical parameters of rats in a high-altitude hypoxic environment from the blood perspective, which can provide an experimental basis for research on the pathogenesis of high-altitude diseases.

## Data Availability

The data that support the findings of this study are available from the corresponding author upon reasonable request.
